# 雷公藤甲素与吉非替尼序贯应用对人肺腺癌细胞H1975存活及凋亡通路的影响

**DOI:** 10.3779/j.issn.1009-3419.2015.10.01

**Published:** 2015-10-20

**Authors:** 鑫宇 张, 皈阳 刘, 浩 刘, 涛 马

**Affiliations:** 1 233000 蚌埠，蚌埠医学院药学系 Department of Pharmacy, Bengbu Medical College, Bengbu 233000, China; 2 100853 北京，中国人民解放军附属医院 Department of Pharmacy, the Affiliated Hospital of Chinese PLA Hospital, Beijing 100853, China

**Keywords:** T790M, H1975, 等效线图法, 雷公藤甲素, 吉非替尼, 凋亡, T790M, H1975, Isobolograms, Triptolide, Geftinib, Apoptosis

## Abstract

**背景与目的:**

表皮生长因子受体(epidermal growth factor receptor, EGFR)酪氨酸激酶抑制剂(tyrosine kinase inhibitors, TKIs)被用于治疗进展性晚期非小细胞肺癌(non-small cell lung cancer, NSCLC), 然而最初接受TKIs治疗有反应的患者, 大都会在10个月左右出现获得性耐药。报告称*EGFR*基因T790M的突变是产生获得性耐药的主要原因, 比例约占50%。本研究旨在探索雷公藤甲素(triptolide, TP)和吉非替尼序贯应用对肺腺癌细胞H1975细胞增殖和凋亡通路的作用。

**方法:**

MTT法检测细胞的增殖。等效线图法和联合指数(combination index, CI)法评估雷公藤甲素和吉非替尼序贯作用的效价。流式细胞术检测细胞凋亡和周期分布, Hoechest 33258染色法检测凋亡形态。化学比色发光法检测Caspases的活性。

**结果:**

等效线图法和联合指数法均显示雷公藤甲素序贯吉非替尼组较其他序贯作用组明显抑制了细胞增殖, 增加了细胞的凋亡。细胞周期分布实验结果显示与吉非替尼序贯雷公藤甲素组主要把细胞抑制在G_0_/G_1_期相比较, 雷公藤甲素序贯吉非替尼组主要把细胞抑制在G_2_/M期。在肺腺癌H1975中, 所有序贯模型组都主要通过活化Caspase-9/Caspase-3来诱导激活细胞凋亡通路。

**结论:**

先用雷公藤甲素再用吉非替尼治疗模式可能是克服T790M突变耐药的一个新选择。

## Abstract (en)

**Background and objective:**

Epidermal growth factor receptor (EGFR) tyrosine kinase inhibitors (TKIs) show promising therapeutic effects in patients with advanced non-small cell lung cancer (NSCLC).However, despite an initial response to TKIs treatment among responsive patients, most inevitably acquire resistance after a progression-free period of about 10 months.The percentage of T790M in TKI acquired-resistant patients in most studies is around 50%.The aim of this study is to assess the effects of the sequential administration of triptolide and geftinib on cell proliferation and apoptosis of lung adenocarcinoma cell H1975.

**Methods:**

A MTT assay was used to measure cell proliferation.The potency of the sequential administration of triptolide and geftinib were determined by isobolograms and combination index (CI).Cell apoptosis and cycle distribution were detected by flow cytometry.The Hoechst 33258 method was used to observe the apoptotic morphology.Chemical colorimetric luminescence was used to measure the caspase activity.

**Results:**

The results of isobolograms and CI showed that the sequential administration of triptolide following geftinib remarkably inhibited cell proliferation and cell apoptosis compared with other sequential administration models.The cycle distribution results indicated that sequential triptolide administration following geftinib blocked the cells in the G_2_/M phase but not in the G_0_/G_1_ phase.The activation of the Caspase-9/Caspase-3 cascade was mainly involved in the apoptotic pathway of lung adenocarcinoma cell H1975 in all sequential administration models.

**Conclusion:**

The triptolide administration following geftinib might be a new therapeutic strategy for lung cancer with T790M mutation after having EGFR-TKIs resistance.

肺癌是当今发生率和死亡率最高的恶性肿瘤之一, 非小细胞肺癌(non-small cell lung cancer, NSCLC)占所有肺癌的80%左右。大部分肺癌患者被确诊时已经属于晚期, 导致患者的5年生存率往往低于10%^[1]^。以表皮生长因子受体(epidermal growth factor receptor, EGFR)为靶点的分子靶向治疗成为肿瘤治疗研究的热点。吉非替尼、厄洛替尼以及国产的埃克替尼等是具有口服活性可逆的小分子酪氨酸激酶抑制剂(tyrosine kinase inhibitors, TKIs), 被广泛应用于晚期进展的NSCLC, 此类药物在胞内和ATP竞争酪氨酸激酶残基的结合位点, 阻止酪氨酸激酶残基自身磷酸化而抑制下游信号的转导, 起到抑制肿瘤生长的目的^[2]^。然而临床观察^[3]^中发现, 虽然TKIs在携带优势突变的患者中显示出较好的反应率, 但获得性耐药却不可避免的尾随而至, 一般出现在最初治疗1年内。在所有的获得性耐药的可能机制中, EGFR 20外显子T790M^[4]^突变约占50%, 是最常见的耐药机制。从发现耐药的那一刻起, 逆转耐药的研究也一直没有停止过。根据TKIs治疗失败的分子机制研究主要有靶向T790M耐药突变开发的第三代不可逆的TKIs等; 各项研究显示在肿瘤无进展生存期、毒副反应以及疾病缓解率等方面都有优势。但真正走向临床服务于患者还需要一个过程。这段时间中, 面对越来越多TKIs治疗失败后仍需治疗或仍有强烈治疗意愿的患者, 需要在已有的治疗药物基础上探索新的治疗模式, 既往相关基础研究发现合理的化疗联合靶向治疗模式在NSCLC *EGFR*突变野生型和敏感型细胞株中均显示有协同作用^[5-7]^。已有临床医生根据TKIs治疗阶段以及治疗过程中患者对TKIs的反应, 借助临床现有的药物和治疗手段, 不断地尝试逆转耐药, 主要尝试有铂类联合紫杉类化疗、化疗联合靶向、化疗后序贯原靶向药物, 以及更换另外一种TKI等模式。虽然大都为小规模尝试治疗的总结, 证据级别不高, 但经验可贵, 值得借鉴^[8, 9]^。

基于上述信息, 我们发现占获得性耐药比例最高的T790M突变人群中, 有关中药及其相关制剂与靶向药物联合应用的研究较少, 因此对于具有较强抗肿瘤活性的中药及其制剂, 值得我们在此方向进行尝试性探索^[10]^。

雷公藤甲素又名雷公藤内酯醇, 是从卫矛科植物雷公藤中提取分离出来的活性最高的含有3个环氧基的二萜内酯化合物, 是雷公藤的主要活性成分之一。其相关效价比雷公藤总苷高100倍-200倍。经药理和临床试验表明其具有免疫抑制、抗炎、抗肿瘤、抗过敏、抗生育等多种生物活性, 体内化学性质稳定, 临床上得以广泛应用, 主要用于治疗自身免疫性疾病和器官移植的排斥反应^[11]^。从20世纪70年代揭开其具有抗肿瘤作用后, 近年来其抗肿瘤作用逐渐为大家所认识并引起广泛重视。有实验结果提示雷公藤甲素及其衍生物雷公藤氯内酯醇对肺腺癌A549细胞具有增殖抑制作用, 可能的机制与诱导细胞凋亡或自噬有关^[12]^, 但在EGFR-TKIs耐药的H1975(EGFR exon 20 T790M-exon 21 L858R)细胞株上未见到相关研究^[13]^。

本研究拟通过体外实验探索雷公藤甲素和吉非替尼在携带T790M突变的NSCLC获得性耐药细胞株H1975上合理的给药模式, 并探讨产生不同结果的机制。一方面进一步完善此方向研究; 另一方面为T790M获得性耐药的后续治疗提供一些值得借鉴的基础理论支持。

## 1 材料与方法

### 1.1 材料

人肺腺癌细胞H1975购自中科院上海细胞生物研究所, MTT、DMSO、吉非替尼、雷公藤甲素均购自Sigma公司。吉非替尼与雷公藤甲素均溶于DMSO中, 分别以40 mmol/L与100 μmol/L过滤分装后于-20 ℃保存, 凋亡细胞染色试剂盒购自普利莱基因技术有限公司, Annexinv-PE/7-AAD细胞凋亡检测试剂盒购自BD公司, DNA含量(细胞周期)检测试剂盒购自南京凯基生物技术有限公司, Caspase-3、Caspase-8、Caspase-9活性检测试剂盒购自R & D Systems。流式细胞仪为BD公司产品, 酶联免疫检测仪为Biocell2010公司产品。

### 1.2 方法

#### 1.2.1 细胞培养

H1975细胞培养于含10%胎牛血清和青霉素与链霉素(终浓度为100 U/mL)的RPMI-1640(Gibco公司)培养液, 常规置于37 ℃、5%CO_2_培养箱中, 根据细胞生长情况进行消化传代, 并取对数生长期细胞实验。

#### 1.2.2 MTT法检测药物对H1975生长作用的影响

通常认为在MTT检测中空白对照组在波长570 nm的吸光度为0.8-1.2时, 不同抑制组与空白组间区分效果较好, 由上述方法确定H1975的铺板数为5, 000个/孔。

##### 1.2.2.1 单药MTT实验

实验前一天晚上取生长状态良好的对数生长期细胞, 胰酶消化计数后完全培养基稀释到上述相应浓度后, 取100 μL到96孔板, 37 ℃、5%CO_2_培养箱中孵育过夜, 待细胞完全贴壁后, 吸弃原培养基, 加入200 μL完全培养基配置好的含不同药物不同浓度的药液, 每个浓度至少6个复孔, 设置空白组和对照组。空白组只加培养液, 对照组不加药物。培养72 h后每孔加用PBS配成的5 mg/mL MTT溶液20 μL, 继续孵育4 h-6 h后小心吸弃上清, 每孔加150 μL DMSO, 置水平摇床上低速振荡10 min, 使结晶物充分溶解, 570 nm波长处测量各孔吸光度^[14, 15]^。分别计算各加药组的抑制率。所有细胞增殖抑制实验均独立重复至少3次, 求得平均抑制率, 细胞存活率=(A_用药组_/A_对照组_)×100%, 细胞抑制率=(1-A_用药组_/A_对照组_×100%, 实验重复3次以上。

##### 1.2.2.2 不同时序给药方案的增殖抑制实验

时序方案分组如下:①雷公藤甲素作用24 h, PBS洗涤1遍, 吉非替尼继续序贯48 h(TG); ②雷公藤甲素联合吉非替尼作用48 h, PBS洗涤1遍, 不含药液的完全培养基继续孵育24 h(T+G); ③吉非替尼作用48 h, PBS洗涤1遍, 雷公藤甲素继续序贯24 h(GT)。不同时序方案下的两药给药浓度根据上述单药MTT的半抑制浓度(half maximal inhibitory concentration, IC_50_)值的比率进行确定, 两药在每种方案下的给药配比剂量分别为各自单药IC_50_的0倍、0.25倍、0.5倍、1倍、2倍、4倍。MTT方法同上述单药实验, 并重复3次以上。

#### 1.2.3 两药时序使用效应的评估

##### 1.2.3.1 Isobolograms等效线图法

通过Steel和Peckham^[16]^的Isobolograms来评价IC_80_(抑制80%细胞生长的药物浓度)水平, Isobolograms法是判定联合用药体外发生协同、相加、拮抗作用的标准统计方法, 适用于毒性机制不明以及多种抗癌因子联合作用下的量效曲线研究。Isobolograms的概念在先前的研究中有过详细的描述^[17]^。

##### 1.2.3.2 联合指数分析

以MTT法测得单药不同药物剂量的抑制率后可以得到细胞的剂量-效应曲线, 采用CalcuSyn分析软件计算联合指数(combination index, CI)。联合指数的公式为CI=(D)1/(Dx)1+(D)2/(Dx)2, 其中(D)1、(D)2分别为联合用药时两药各自的浓度, Dx为联合用药达fa时, 单药抑制率也达到fa时所需要的药物浓度, Dx=Dm[fa/(1-fa)]1/m, fa表示一定浓度的两药联合用药达到的抑制率。通过软件输入单药的剂量及抑制率可得到Dm值和m值, 再经上述联合指数计算公式就可得到某种联合用药方案的效应, CI < 1表示此方案具有协同效应^[18]^。

#### 1.2.4 流式细胞仪Annexin V/7-AAD双染色法检测不同给药方案下H1975细胞凋亡

实验前一天晚上取生长状态良好的对数生长期细胞, 胰酶消化计数后完全培养基稀释到1×10^6^个/mL, 取500 μL至25 cm培养瓶中, 加完全培养基至5 mL, 37 ℃、5%CO_2_培养箱中孵育过夜, 待细胞完全贴壁后, 吸弃原培养基, 加入5 mL完全培养基配置好的含不同药物浓度的药液。到达作用时间后, 不含EDTA的胰酶消化收集细胞, PBS洗涤2次, 计数收集5×10^5^个-1×10^6^个细胞, 加入500 μL-1, 000 μL的Binding Buffer重悬细胞调整细胞浓度到1×10^6^个/mL。从以上细胞悬液中吸取100 L转移到5 mL流式上样管中, 分别加入5 μL 7-AAD和PE-Annexin V染液, 室温避光反应至少15 min, 最后向每支流式管中加入300 μL-400 μL Binding Buffer, 1 h内上机检测, 激发波长Ex=488 nm, 发射波长Em=578 nm, 每次实验均需在正常细胞组中设立3组对照:①不加染料组; ②7-AAD单染组; ③PE-Annexin V单染组。实验重复3次。实验分组如下:①对照(无药物作用)72 h组(N); ②雷公藤甲素单独作用72 h组(T); ③吉非替尼单独作用72 h组(G); ④雷公藤甲素作用24 h, PBS洗涤1次后序贯吉非替尼48 h组(TG); ⑤雷公藤甲素联合吉非替尼48 h, PBS洗涤1次后序贯不含药液的完全培养基继续孵育24 h组(T+G); ⑥吉非替尼作用48 h, PBS洗涤1次后序贯雷公藤甲素24 h组(GT)。每种方案下的给药剂量分别为MTT单药实验中的IC_50_。

#### 1.2.5 Hoechst 33258 DNA染色检测凋亡

实验分组同上, 收集各组细胞到1.5 mL EP管中, 4%多聚甲醛重悬细胞沉淀固定, 1, 000 rpm低速离心, 吸弃固定液, PBS洗涤1次, 将少量细胞悬液滴到载玻片上, 涂布, 风干; 使用配制好的Hoechst 33342工作液, 重悬细胞, 室温孵育10 min以上; 吸弃染色液, PBS洗涤1次, 封片。每组细胞至少涂片5张, 每张细胞涂片在Olympus荧光显微镜200倍视野下观察8个不同视野, 实验重复3次。

#### 1.2.6 流式细胞仪检测不同给药方案下H1975细胞周期分布

实验分组和给药剂量同凋亡实验, 到达作用时间后, 胰蛋白酶-EDTA消化液消化收集细胞, PBS洗涤细胞1次, 计数调整细胞浓度为1×10^6^个/mL, 70%冰乙醇固定, 4 ℃保存, 染色前PBS洗去固定液, 加100 μL RNaseA 37 ℃水浴30 min, 再避光加入400 μL PI染液混匀, 4 ℃避光30 min, 上机检测, 记录激发波长488 nm处红色荧光, 检测细胞周期分布。

#### 1.2.7 Caspase活性检测

实验分组和给药剂量同凋亡实验, 收集2×10^6^个-6×10^6^个细胞, PBS洗涤2次, 加50 μL-150 μL冰冷的Lysis Buffer(25 μL/1×10^6^细胞), 冰上孵育10 min, 离心10, 000 g、3 min, 将上清转移至新的EP管, 取少量上清(5 μL), Braford法测定其中的蛋白浓度; 吸取50 μL含100 g-200 g蛋白裂解上清; 如体积不足50 μL用Lysis Buffer补足至总体积50 μL, 加入50 μL的2×Reaction Buffer(注意:使用前每50 μL 2×Reaction Buffer加入0.5 μL DTT, Reaction Buffer要一一对应); 根据实验目的加入5 μL Caspase-3(DEVD-AFC)、Caspase-8(IETD-AFC)或者Caspase-9(LEHD-AFC), 并于37 ℃避光孵育2 h。酶标仪λ=405 nm测定其吸光值。通过计算OD_诱导剂_/OD_阴性对照_的倍数来确定凋亡诱导剂组Caspase的活化程度。同时设置空白组(50 μL Lysis Buffer+50 μL 2×Reaction Buffer+5 μL底物)以及不含底物的阴性组(50 μL含蛋白Lysis Buffer+50 μL 2×Reaction Buffer)。

### 1.3 统计学方法

采用SPSS 13.0软件进行统计学分析, 各组间差异分析采用*One-way ANOVA*检验, 以*P* < 0.05为差异有统计学意义。

## 2 结果

### 2.1 单药MTT对H1975生长的影响

雷公藤甲素和吉非替尼作用72 h对H1975细胞抑制作用的IC_50_值分别为(26.9±3.8)nmol/L、(9.9±0.9)μmol/L, 并且呈浓度依赖性抑制H1975的生长(图 1)。

**1:**
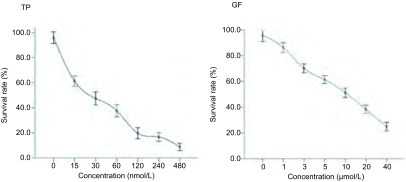
不同浓度的雷公藤甲素(左)与吉非替尼(右)作用72 h对H1975细胞生长的影响(*n*=3) Effects of triptolide (left) and gefitinib (right) on the proliferation of H1975 cells for 72 h (*n*=3)

### 2.2 不同时序给药方案对H1975增殖抑制作用

根据吉非替尼和雷公藤甲素单药的MTT结果, 近似取IC_50_的整数值, 吉非替尼为10 μmol/L, 雷公藤甲素为25 nmol/L。按照上述设计的3种时序给药方案和药物配比剂量进行MTT试验, 得到的结果见图 2。其中A、B、C分别代表雷公藤甲素序贯吉非替尼组、雷公藤甲素联合吉非替尼组以及吉非替尼序贯雷公藤甲素组。纵坐标显示的是H1975的存活率, 横坐标对应的是雷公藤甲素和吉非替尼的浓度配比。依据3种给药模型中两药单药量效曲线可进一步绘制IC_80_下不同方案等效线图, 从而评价模型之间的优劣。同时可以进行3种模型联合用药指数CI的分析。

**2:**
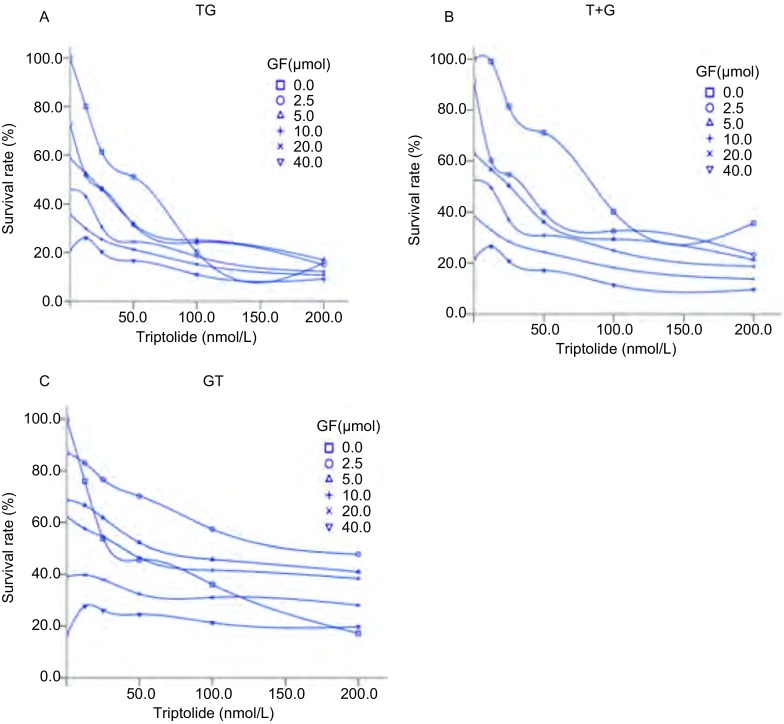
吉非替尼和雷公藤甲素在H1975细胞株中的时序依赖关系(*n*=3)。A:雷公藤甲素24 h后吉非替尼48 h; B:雷公藤甲素联合吉非替尼48 h序贯24 h不含药完全培养基; C:吉非替尼48 h序贯雷公藤甲素24 h。 Schedule dependence of the interaction between Gefitinib and Triptolide in H1975 (*n*=3).A:Pretreated with triptolide 24 h, followed by gefitinib for 48 h; B:Treated concomitantly with gefitinib and triptolide for 48 h and incubated in drug-free medium for 24 h; C:Pretreated with gefitinib 48 h, followed by triptolide for 24 h.TG:Triptolide followed by Gefitinib; T+G:Triptolide plus Gefitinib; GT:Gefitinib followed by Triptolide.

### 2.3 不同时序模型给药方案效果的评价

#### 2.3.1 等效线图法

依据3种给药模型中吉非替尼和雷公藤甲素单药量效曲线绘制IC_80_下3种方案的等效线图模型(图 3)。同时进行3种模型联合用药指数CI的分析(表 1)。图 4中A、B、C分别代表雷公藤甲素24 h序贯吉非替尼组48 h、雷公藤甲素联合吉非替尼组48 h以及吉非替尼48 h序贯雷公藤甲素24 h组的等效线图, 各图中横纵坐标数值1分别代表每种模型下吉非替尼或雷公藤甲素单药作用后的IC_80_值。A图中固定吉非替尼剂量后, 变换雷公藤甲素的剂量得到的5个IC_80_等效点中3个点落在IC_80_等效口袋区域的左侧, 2个点处于等效区域的左侧边缘位置, 说明雷公藤甲素序贯吉非替尼组主要表示为协同作用或接近强相加作用。B图中5个IC_80_等效点皆处于IC_80_等效口袋区域内, 且大都处于区域靠右侧位置, 说明雷公藤甲素联合吉非替尼主要表示为相加作用。C图中5个IC_80_等效点皆处于IC_80_等效区域右侧且远离等效区域, 说明吉非替尼序贯雷公藤甲素主要为弱相加或拮抗作用。

**3:**
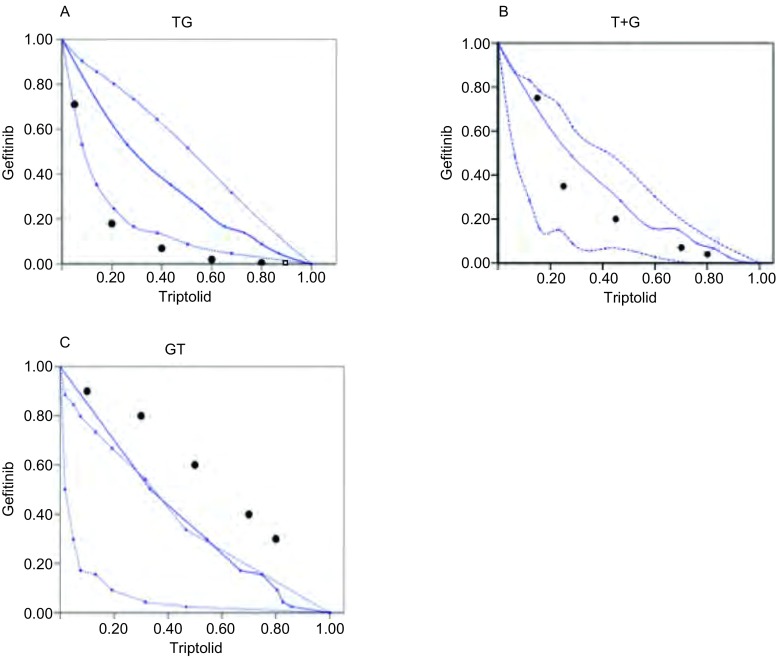
雷公藤甲素与吉非替尼时序作用下的等效线图(*n*=3)。A:雷公藤甲素24 h后吉非替尼48 h; B:雷公藤甲素联合吉非替尼48 h序贯24 h不含药完全培养基; C:吉非替尼48 h序贯雷公藤甲素24 h。数据为3次独立实验平均值。 Isobologram of triptolide in combination with gefitinib (*n*=3).A:Pre-treated with triptolide 24 h, followed by gefitinib for 48 h; B:Treated concomitantly with gefitinib and triptolide for 48 h and incubated in drug-free medium for 24 h; C:Pretreated with gefitinib 48 h, followed by triptolide for 24 h.

**1:** 雷公藤甲素序贯吉非替尼组的联合用药指数结果 The result of CI to pretreated with triptolide followed by gefitinib

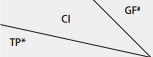	40	15	10	5	2.5
6.25	0.37	0.58	0.9	0.87	1.03
12.5	0.45	0.53	0.67	0.79	1.18
25	0.39	0.55	0.78	0.59	0.87
50	0.48	0.65	0.76	0.64	0.57
100	0.59	0.71	0.83	0.56	0.52
Average	0.46	0.60	0.79	0.69	0.83
^*^:nmol/L; #:μmol/L; CI:combination index; TP:triptolide; GF:gefitinib.					

**4:**
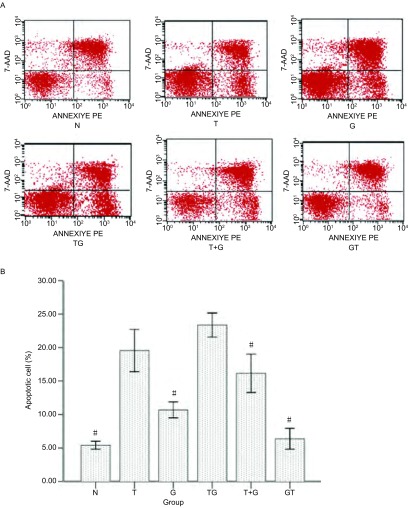
雷公藤甲素和吉非替尼单药或序贯作用72 h诱导的H1975凋亡(*n*=3)。A:N:正常培养; T:雷公藤甲素单药; G:吉非替尼单药; TG:雷公藤甲素序贯吉非替尼; T+G:雷公藤甲素联合吉非替尼; GT:吉非替尼序贯雷公藤甲素。B:与正常培养组对比其他组的凋亡率。#:与T/TG组比较, *P* < 0.05。 The effects of triptolide and gefitinib alone and in the sequential exposure schedules for 72 h on cell apoptosis of H1975 (*n*=3).A:N:Without treatment; T:Triptolide alone; G:Gefitinib alone; TG:Triptolide followed by Gefitinib; T+G:Triptolide plus Gefitinib; GT:Gefitinib followed by Triptolide.B:Relative apoptosis levels to control.#:Compared with T/TG group, *P* < 0.05.

#### 2.3.2 中位线效应法

对3种给药模型得到的原始数据按照联合用药指数法进行分析采用CalcuSyn软件计算联合用药的CI值, 具体结果见表 1-表 3。从CI的概念来讲, CI > 1说明药物间为拮抗作用, CI=1说明药物间为相加作用, CI < 1说明药物间为协同作用。CI值越小, 协同的效应越强烈。在实际实验中, 通常认为CI < 0.3表示强烈协同效应, 0.3 < CI < 0.7为中度协同效应, 0.7 CI < 1.0为较弱的协同效应。表 1结果显示大部分值小于1, 在吉非替尼低剂量的组别中个别值接近1或略高于1, 说明雷公藤甲素序贯吉非替尼组主要表现为强协同或中度协同作用。表 2结果显示大部分结果在1附近, 在吉非替尼低剂量组别高于1的现象, 说明雷公藤甲素联合吉非替尼主要表现为相加作用。表 3结果显示大部分值远远超过1, 表示吉非替尼序贯雷公藤甲素主要表现为拮抗作用。

**2:** 雷公藤甲素联合吉非替尼的联合用药指数结果 The result of CI to treated gefitinib combination with triptolide

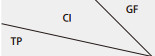	40	15	10	5	2.5
6.25	0.89	0.93	0.98	1.09	1.13
12.5	0.92	0.87	1.01	0.96	1.09
25	1.07	0.91	1.23	1.04	0.99
50	1.13	0.94	1.08	1.12	1.21
100	1.24	1.07	1.17	1.28	1.32
Average	1.05	0.94	1.10	1.09	1.15

**3:** 吉非替尼序贯雷公藤甲素的联合用药指数结果 The result of CI to pretreated with gefitinb followed by triptolide

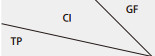	40	15	10	5	2.5
6.25	1.39	1.48	1.59	1.82	1.93
12.5	1.35	1.35	1.55	1.62	1.82
25	1.58	1.47	1.85	1.78	1.80
50	1.84	1.70	1.90	2.06	2.22
100	1.88	1.76	1.92	2.15	2.26
Average	1.61	1.55	1.76	1.89	2.01

### 2.4 Annexin V/7-AAD双染色法检测吉非替尼和雷公藤甲素诱导H1975细胞的凋亡

采用流式细胞仪分析方法进一步确定雷公藤甲素序贯吉非替尼是否导致最高的凋亡率。共设计6种给药方案, 其中包括空白对照组以及雷公藤甲素和吉非替尼单药组(图 4A), 结果发现与正常组相比, 10 μmol吉非替尼单药(10.71±0.51)以及吉非替尼序贯雷公藤甲素(6.41±0.64)方案均未引起H1975出现明显的凋亡现象, 吉非替尼联合雷公藤甲素同吉非替尼单药以及雷公藤甲素序贯吉非替尼与空白组比较皆诱导产生明显凋亡(*P* < 0.05), 而且雷公藤甲素单药(19.63±1.31)与雷公藤甲素序贯吉非替尼(23.41±0.74)方案较雷公藤甲素联合吉非替尼方案(16.15±1.21)在诱导凋亡结果上也具有明显差异(*P* < 0.05)。但雷公藤甲素单药和雷公藤甲素序贯吉非替尼两种方案间未能得出有统计学意义的结果, 数值上雷公藤甲素序贯吉非替尼组略显优势(图 4B)。

### 2.5 药物处理后Hoechest 33258染色结果

药物诱导凋亡的H1975细胞, Hoechest 33258染色后显示蓝染的凋亡小体和细胞核。与完整、圆形、大型的细胞核相比, 单个凋亡小体呈细小不规则颗粒状, 同一细胞内(红色箭头指示处)可出现大小不等的多个凋亡小体(图 5)。

**5:**
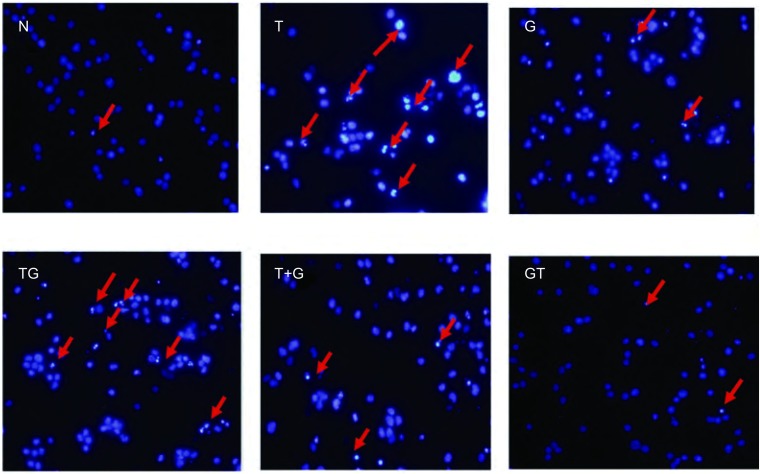
不同方法处理72 h后H1975凋亡形态改变(*n*=3)。红色箭头所指为凋亡细胞 The apoptosis of H1975 in dealing with different methods after 72 h (*n*=3).The red arrow refers to apoptotic cells.

### 2.6 不同给药方式对细胞周期的影响

给药剂量和实验分组同细胞凋亡实验, 不同作用方式下的结果都以正常为基准进行比较, 流式图结果见图 6, 由统计结果可以看出, 吉非替尼单药主要将细胞抑制在G_0_/G_1_期(59.82±3.41)(*P* < 0.05), 而单药雷公藤甲素作用后, G_2_/M期的细胞明显增多(45.68±1.51)(*P* < 0.05), 同样在吉非替尼序贯雷公藤甲素方案中G_0_/G_1_期细胞高达(61.51±3.92)(*P* < 0.05), 而雷公藤甲素序贯吉非替尼组G_0_/G_1_期细胞仅占(33.73±2.81), G_2_/M期细胞比例则为(42.95±1.39)(*P* < 0.05)。由此可见吉非替尼单药或吉非替尼序贯雷公藤甲素组主要诱导G_0_/G_1_期阻滞, 而雷公藤甲素单药或雷公藤甲素序贯吉非替尼组主要诱导G_2_/M期阻滞。

**6:**
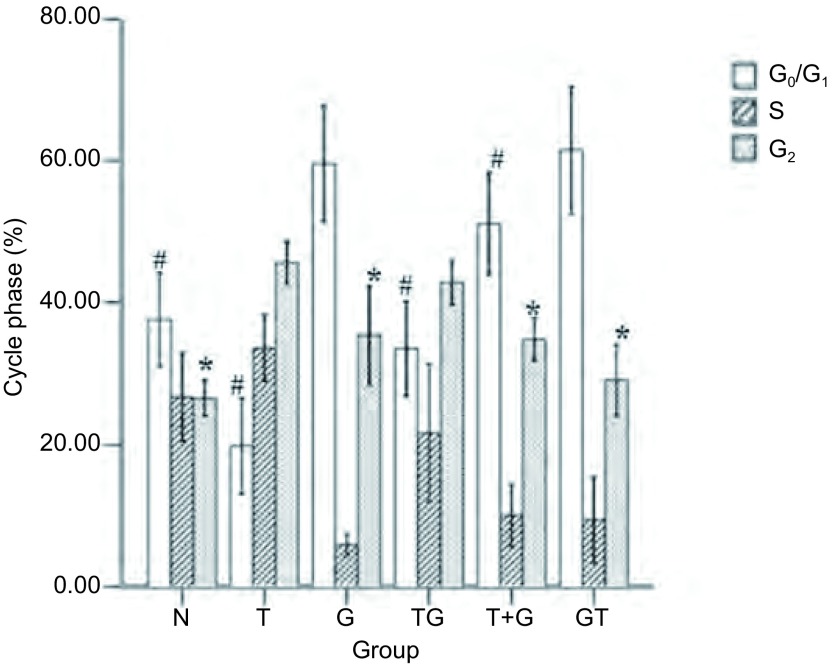
吉非替尼与雷公藤甲素联合作用于H1975细胞时的细胞周期分布(*n*=3)。与正常培养组对比其他组的周期分布。#:与GT/G组比较, *P* < 0.05。^*^:与T/TG组比较, *P* < 0.05。 Cell cycle distribution of H1975 cells exposed to gefitinib and triptolide in different sequences (*n*=3).Relative cell cycle distribution levels to control.#:Compared with GT/G group, *P* < 0.05;^*^:Compared with T/TG group, *P* < 0.05.

### 2.7 不同给药方式下Caspase-3、Caspase-8、Caspase-9的活性情况

由方法中可知Caspase的活化程度可以通过计算OD_诱导剂_/OD_阴性对照_的倍数来确定。统计结果发现与其他组相比, 雷公藤甲素单药组同雷公藤甲素序贯吉非替尼组Caspase-3的活性分别为(2.23±0.36)和(2.51±0.21)(*P* < 0.05), Caspase-9的活性分别为(2.61±0.31)和(2.11±0.23)(*P* < 0.05), 而所有组Caspase-8的活性无统计学差异(图 7)。

**7:**
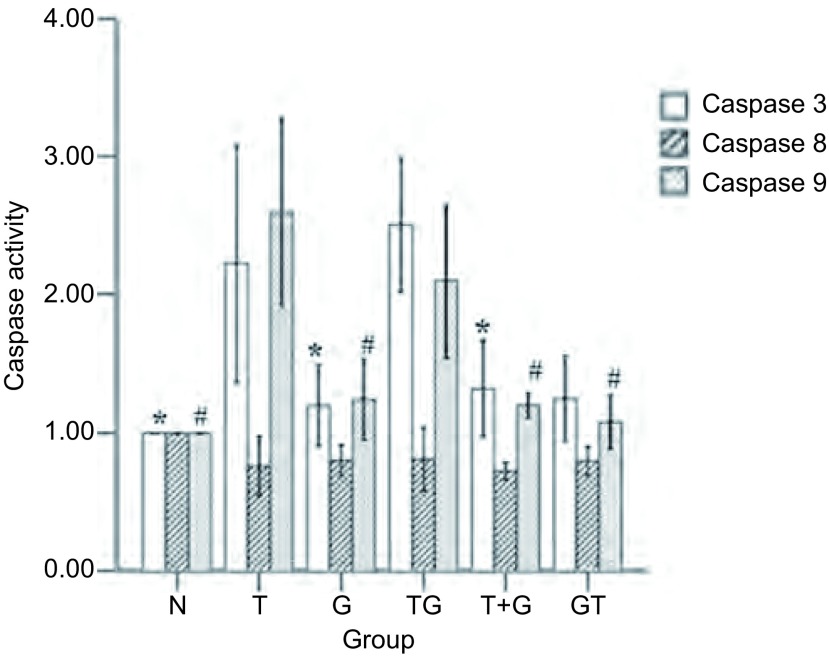
吉非替尼与雷公藤甲素联合下Caspase的活化情况(*n*=3)。^*^:与T/TG组比较, *P* < 0.05。#:与T/TG组比较, *P* < 0.05。 The activities of caspases to gefitinib and triptolide in different sequences (*n*=3).^*^:Compared with T/TG group, *P* < 0.05;#:Compared with T/TG group, *P* < 0.05.

## 3 讨论

EGFR的异常表达和活化在肿瘤的发生发展中起着重要的作用, 使EGFR成为肿瘤治疗的理想靶位, 第一代TKIs通过抑制EGFR及其下游信号磷脂酰肌醇三羟基激酶/蛋白激酶B(phosphatidylinositol-3-kinase and protein kinase B, PI3K/Akt)和丝裂原活化蛋白激酶/细胞外信号调节激酶1/2(mitogen-activated protein kinases/extracellular signal-reglated kinases, MAPK/Erk1/2)传导, 从而然抑制肿瘤细胞生长和增殖, 诱导凋亡, 大大提高了晚期*EGFR*突变患者的无疾病生存时间和缓解率, 但大部分患者或早或晚都将出现耐药。其中20外显子790位T突变为M(蛋白水平表现为苏氨酸转变为甲硫氨酸)是肿瘤对EGFR-TKIs获得性耐药的重要原因, 约占50%。H1975细胞为携带21外显子敏感性突变的双突变耐药细胞株, 双突变造成的ATP亲和力的恢复使得加大的治疗窗重新关闭, 表现为对TKIs的获得性耐药。我们经过反复实验总结出了得到吉非替尼和雷公藤甲素单药作用72 h对H1975细胞抑制作用的IC_50_值分别为(9.93±0.91)mol/L和(26.91±3.82)nmol/L, 在以往报道^[19]^的范围之内。TKIs耐药后的临床治疗一直处于探索阶段, 比如在耐药后进行铂类联合紫杉类化疗、化疗联合靶向治疗、化疗后序贯原靶向治疗以及更换另外一种TKIs药物的尝试均有报道^[20]^。但从以上相关报道我们发现逆转T790M导致的耐药的探索过程中有关中药及其相关制剂与靶向药物联合应用的研究较少, 而雷公藤甲素近些年来被广泛报道应用于不同种类的癌症治疗中, 同样在NSCLC方面, 无论在体外和体内, 可以明显抑制相应肿瘤细胞和移植瘤。因此探究雷公藤甲素和TKIs合理治疗模式对于探索T790M导致的获得性耐药有一定的参考价值。

联合用药实验中的“协同”是指“1+1 > 2”的效果, 针对携带T790M突变的H1975细胞株, 我们设计了3种序贯方案来考察哪种方案的疗效最佳。常用于评估两药联合效应的统计学方法有Isobolograms模型、Bliss additivism模型及CI方法。Isobolograms模型和CI方法均是基于IC_50_进行计算的^[21]^。本实验中的数据达到了使用Isobolograms模型和CI方法的条件。所以我们同时选择这两种方法来体外评价上述3种给药方案对H1975存活的影响。

研究结果显示, 先用雷公藤甲素24 h后序贯吉非替尼48 h组中5个IC_80_等效点中4个点落在IC_80_等效口袋区域的左侧, 相对于雷公藤甲素联合吉非替尼48 h组(5个点皆处于IC_80_等效口袋区域内, 指示为相加作用)以及吉非替尼48 h后序贯雷公藤甲素24 h组(5个IC_80_等效点皆处于IC_80_等效区域右侧, 指示为弱相加或拮抗作用)表示为协同作用或接近强相加作用。表 1-表 3中的CI值同样也证实了Isobolograms模型的结论, 即雷公藤甲素序贯吉非替尼组较其他两种给药方式具有协同增效作用。

目前有报道^[22]^称吉非替尼低浓度仅引起细胞生长的抑制, 只有高浓度才会导致细胞的凋亡, 并且凋亡主要经死亡受体途径诱导。而雷公藤甲素抑制NSCLC可能的方式为干扰细胞周期, 激活caspase信号通路、抑制血管内皮生长因子或抑制NF-κB的活性有关, 但未见有明确结论^[23]^。因此我们在细胞毒性数据及3种序贯给药方式的基础上, 增加空白组和单药组, 对不同方案下细胞死亡方式中的凋亡途径进行初步研究, 探讨不同用药组下细胞周期分布情况, 观察不同凋亡途径的交叉和影响, 进一步解释不同方案间存在的疗效差异现象。流式细胞仪分析方法检测凋亡实验结果显示:雷公藤甲素单药与雷公藤甲素序贯吉非替尼在诱导凋亡结果上较其它组具有明显差异, 数值上雷公藤甲素序贯吉非替尼组略显优势。而单药吉非替尼和吉非替尼序贯雷公藤甲素组, 未诱导出明显凋亡。细胞周期分布实验中单药雷公藤甲素和雷公藤甲素序贯吉非替尼组, 细胞主要被抑制在G_2_/M期, 而单药吉非替尼和吉非替尼序贯雷公藤甲素组, 细胞主要被抑制在G_0_/G_1_期。Caspase活化结果显示单药雷公藤甲素和雷公藤甲素序贯吉非替尼组, Caspase-3、Caspase-9明显活化, 而Caspase-8与对照组相比, 不仅未被诱导活化, 而且活性均有一定程度的抑制。

研究发现化疗与EGFR-TKIs联合应用于不同NSCLC细胞, 只有细胞EGFR磷酸化(pEGFR)水平增加者才能从后续的吉非替尼治疗中获益, 而与EGFR是否存在突变或扩增无关, 也就是说先雷公藤甲素后吉非替尼的EGFR和ERK磷酸化水平会明显增高, 明显提高了后续吉非替尼的疗效。同时吉非替尼为细胞周期非特异性药物, 使大量细胞在雷公藤甲素诱导的G_2_/M期死亡。而吉非替尼与雷公藤甲素同时应用或吉非替尼后序贯雷公藤甲素时, 吉非替尼把细胞大量抑制在G_0_/G_1_期, 而低浓度的雷公藤甲素主要把细胞阻滞在S期, 高浓度时主要把细胞阻滞在G_2_/M期, 被吉非替尼大量抑制在G_0_/G_1_期的细胞导致雷公藤甲素的疗效大打折扣, 而吉非替尼导致的EGFR和ERK磷酸化下调作用也不能被同时或序贯应用的雷公藤甲素逆转, 结果说明上述两种方案未能增强细胞抑制作用, 可能与吉非替尼把细胞大量抑制在G_0_/G_1_期和雷公藤甲素细胞周期选择性抑制以及逆转EGFR和ERK磷酸化下调失败有关。Caspase全称为含半胱氨酸的天冬氨酸蛋白水解酶, 是一组存在于细胞质中、具有类似结构的蛋白酶。他们的活性位点均包含半胱氨酸残基, 能够特异性地切割靶蛋白天冬氨酸残基后的肽键, 负责选择性地切割某些蛋白质, 从而造成细胞凋亡。Caspase-8、Caspase-9为起始者胱天蛋白酶, Caspase-3为效应者胱天蛋白酶。Caspase-8主要作用于死亡受体途径, Caspase-9则主要存在于线粒体途径中。二者最终都通过活化下游的Caspase-3来造成细胞凋亡。结果显示, 与空白组比较, 各用药方案皆主要通过线粒体途径中的Caspase-9前体活化从而激活Caspase-3来诱导细胞凋亡, 而且雷公藤甲素序贯吉非替尼组Caspase-3、Caspase-9的活化程度远高于吉非替尼联合雷公藤甲素组以及吉非替尼序贯雷公藤甲素组, 却低于雷公藤甲素单药组。然而流式结果显示雷公藤甲素序贯吉非替尼组的凋亡率反而略高于雷公藤甲素单药组。值得我们注意的是, 所有实验组的Caspase-8的活性并未有统计学差异, 这引发了我们的思考:是否存在两药联合后, 吉非替尼激活血管内皮生长因子或NF-κB的活性, 从而提高了雷公藤甲素的药理学活性, 而这些结果同凋亡之间存在什么样的关系仍值得我们进一步实验继续探索。

中医药是我国的国粹, 几千年来积累了丰富临床应用实践经验, 为深入研究天然药物提取物的抗肿瘤特性提供了很好的技术和理论支持, 雷公藤甲素是一种传统中药, 在肺癌的治疗过程中仍具有很大潜力等待挖掘。本研究在吉非替尼耐药的H1975细胞株上发现雷公藤甲素序贯吉非替尼与吉非替尼联合雷公藤甲素及吉非替尼序贯雷公藤甲素相比具有协同增效作用, 为临床晚期TKIs耐药后T790M突变的患者的治疗提供了一条很好的临床思路, 尤其为体力状态较好的患者带来了一丝希望。但序贯方案的优化和给药剂量的详细确定仍有待进一步体内和临床实验的研究。在死亡相关的凋亡通路中, 不同给药方案主要都是通过线粒体途径诱导细胞凋亡的, 同时雷公藤甲素序贯吉非替尼组诱导凋亡现象仍值得深入研究。
